# Smart and UV-Resistant Edible Coating and Films Based on Alginate, Whey Protein, and Curcumin

**DOI:** 10.3390/polym16040447

**Published:** 2024-02-06

**Authors:** Atcharaporn Botalo, Thitirat Inprasit, Sarute Ummartyotin, Kittipong Chainok, Suteera Vatthanakul, Penwisa Pisitsak

**Affiliations:** 1 Department of Materials and Textile Technology, Faculty of Science and Technology, Thammasat University, Pathum Thani 12121, Thailand; pang120842@gmail.com (A.B.); thi_inp@tu.ac.th (T.I.); sarute@tu.ac.th (S.U.); kc@tu.ac.th (K.C.); 2 Department of Food Science and Technology, Faculty of Science and Technology, Thammasat University, Pathum Thani 12121, Thailand; emmesuteera@gmail.com; 3 Center of Excellence on Petrochemical and Materials Technology, Chulalongkorn University, Bangkok 10330, Thailand

**Keywords:** alginate, active packaging, edible coating, edible film, curcumin, smart packaging, whey protein, UPF rating, UV-blocking film

## Abstract

In this work, smart edible coating and films with excellent UV barrier properties were prepared from alginate, whey protein isolate, and curcumin. The primary focus of this investigation centered on assessing the impact of whey protein and curcumin on the physical and functional properties of the alginate films. Whey protein reduced the film transparency while simultaneously enhancing the hydrophobicity and antioxidant properties of the alginate film. Curcumin imparted a yellow hue to the film, consequently decreasing the transparency of the film. It also substantially improved hydrophobicity, antioxidant activity, and UV-blocking efficiency within the films. Remarkably, curcumin demonstrated a significant reduction in the water vapor transmission rate of the film. For the preservation of apples, a higher concentration of curcumin was required, which effectively suppressed the respiration rate and moisture loss post-harvest, resulting in an extended shelf-life for the apples. As a result, the coated apples exhibited significantly reduced enzymatic browning and weight loss in comparison to their uncoated counterparts. Furthermore, these curcumin-containing films underwent a reversible color change from orange to red when exposed to ammonia vapor. This attribute highlights the potential of the developed coating and film as a smart, active food packaging solution, particularly for light-sensitive food products.

## 1. Introduction

Edible coatings and films used in food packaging have garnered significant attention for their ability to reduce crop losses by safeguarding the quality of post-harvested products. These coatings and films are composed of edible components, including polysaccharides, proteins, and lipids [1], and often serve multiple purposes. Their diverse functions include protection against mechanical stress, the regulation of gas and water transport, and the provision of barriers to both UV and visible light [2,3,4]. Recently, smart packaging has emerged as a new generation of packaging that is capable of not only extending the shelf-life of products but also providing the user with information on the quality of the product. Therefore, smart packaging improves the traceability of the products while passing through the supply chain [5]. Specifically, the age, temperature, and freshness of the package contents can be monitored by integrating an appropriate indicator or sensor inside or outside the packaging. For example, thermochromic ink has been used to indicate the temperature of beer in a metal or glass container [6], while an edible, anthocyanin-loaded film has been used to exhibit a color change in response to a change in pH caused by the deterioration of stored food (salmon) [7]. In another study, two pH-sensitive dyes extracted from blueberry and red grape skin pomace were incorporated into edible films to provide visible color changes due to the spoilage of stored chicken meat [8].

Apples are one of the most widely consumed fruits globally. Edible coatings reduce the respiration rate of apples by minimizing the exchange of O_2_, CO_2_, and H_2_O with the external environment, in addition to mitigating enzymatic browning caused by ethylene production. The integration of bioactive agents, such as antimicrobial agents, anti-browning agents, or antioxidants, is highly desirable, often taking the form of essential oils and botanical extracts [4]. A variety of edible coating formulations can be found in the literature, encompassing chitosan, proteins, lipids, starches, and polysaccharides [4,9]. Commercially available edible coatings suitable for apples include fatty acid esters, carnauba wax, paraffin wax, wood resin, oxidized polyethylene wax, and shellac [4].

Alginate, a polysaccharide derived from brown algae, exhibits good film-forming properties; its structural units consist of β-d-mannuronic acid and α-l-guluronic acid. Alginate is inherently hydrophilic and thus is not suited to controlling water vapor migration. Instead, it functions as a sacrificial moisture agent, allowing moisture to evaporate from the film rather than the food surface [2]. Alginate has been effectively utilized to preserve the post-harvest quality of various fruits, including tomatoes, peaches, sweet cherries, and plums [10].

Whey protein, a by-product of cheese production, contains various components like α-lactalbumin, β-lactoglobulin, serum albumin, lactoperoxidase, immunoglobulins, and lactoferrin, endowing it with high nutritional value [11,12]. When combined with substances like bee waxes, ascorbic acid, and cysteine, whey protein concentrate has proven effective in mitigating browning in fresh-cut apples. It is worth noting that whey protein does not significantly reduce the weight loss of fresh-cut apples [13]. Whey protein nanofibers (WPNF) can be produced through the self-assembly of whey protein isolate with trehalose, as demonstrated by Feng et al. (2018), leading to increased hydrophobicity and enhanced antioxidant properties of the WPNF-based films, along with improved smoothness and uniformity [14].

Curcumin, a naturally occurring colorant, is a hydrophobic polyphenol compound extracted from turmeric roots. It was proposed that curcumin would enhance UV protection and reduce the sensitivity of the film to moisture. Furthermore, the well-documented antioxidant, antimicrobial, anti-inflammatory, anti-cancer, and anti-viral properties of curcumin make it a valuable asset for food preservation [15,16]. Curcumin-loaded electrospun zein nanofibers have demonstrated antifungal properties and conferred nutraceutical benefits to the coated apples [17].

When producing edible films, an interesting approach is to incorporate not only polysaccharides to act as gas barriers but also various proteins to enhance the mechanical properties of the film and hydrophobic additives such as lipids to reduce the water permeability [2]. Therefore, the present study is aimed at enhancing the functional and mechanical properties of edible coatings and films by incorporating the polysaccharide alginate along with whey protein isolate and the natural dye curcumin to achieve a balance in the overall film properties. Moreover, the preservation efficacy of the as-fabricated coatings is assessed by application to whole apples. To this end, the weight loss and enzymatic browning are each monitored during incubation.

## 2. Materials and Methods

### 2.1. Materials

Sodium alginate (91%) was obtained from Loba Chemie (Mumbai, India). The whey protein isolate (WPI) used herein was of 90% purity. Curcumin (CUR, 98%) was obtained from Acros Organics (Mumbai, India). 2,2-diphenyl-2-picrylhydrazyl (DPPH, 95%) was purchased from Thermo Fisher Scientific (Geel, Belgium). Ethanol (98.0%), glycerol (GLY, 99.5%), and citric acid (CA, 99%) were of analytical grade and obtained from commercial sources.

### 2.2. Preparation of Edible Films

WPI (1 g) was dissolved in 150 mL of distilled water and stirred mechanically at 70 °C for 10 min. Subsequently, sodium alginate (5 g), GLY (4 g), and CA (0.3 g) were added to the solution. After 50 min of continuous stirring, the solution (80 mL) was cast onto a 20 × 20 × 4.5 cm (W × L × D) Teflon mold. The cast solution was air-dried at 25 °C for 24 h to form a biopolymer film denoted as SW. The film was then subjected to heat treatment for 5 min at 145 °C in a hot-air oven (Memmert, UE400, Schwabach, Germany). A pure alginate film (denoted as SA) was also prepared using the same technique but without the addition of WPI.

For the CUR-loaded films, firstly, Cur (0.2 or 0.5 g) was dissolved in 20 mL of the water/ethanol mixture (1:3 volume ratio) and stirred until the solution became homogeneous. WPI (1 g) was dissolved in 130 mL of distilled water at 70 °C and stirred for 5 min. The Cur solution was then mixed with the WPI solution and stirred for 10 min. Alginate (5 g) and Gly (4 g) were then added to this solution and stirred for 15 min. Finally, CA (0.3 g) was added to the solution and stirred for 50 min. The film solution was then cast onto a 20 × 20 × 4.5 cm (W × L × D) Teflon mold. The cast solution was then air-dried at 25 °C for 24 h to form a film. The notations SWC1 and SWC2 referred to the films prepared using 0.2 g Cur and 0.5 g Cur, respectively.

### 2.3. Physical Appearance

The thickness of the prepared films was measured using a digital micrometer. The measurements were taken in triplicate. The measurement of haze (a measure of the ratio of diffuse light to the total light transmitted by a specimen) was conducted using a spectrophotometer (UltraScan Pro, Hunter Lab, Reston, VA, USA).

### 2.4. Optical Microscopy

The surfaces of the films were examined by using an optical microscope (Leica DM750 M, Chicago, IL, USA) at ×20 magnification.

### 2.5. Mechanical Properties

Tensile tests were conducted using a universal testing machine (Lloyd Instruments LS 2.5, Bognor Regis, UK) with an extension speed of 100 mm/min and an initial gauge length of 50 mm. All samples were cut to the dimensions of 70 mm in length and 30 mm in width. At least five specimens were tested for each reported value.

### 2.6. Water Solubility Test

First, the film (0.3 g) was weighed and subsequently immersed in 20 mL of distilled water at 25 °C for 24 h. After that, the film was dried at 80 °C for 3 h, and then the water solubility was calculated using Equation (1):

(1) $$\mathrm{Water}\;\mathrm{solubility}\;(\%)=\frac{W_{0}-W_{f}}{W_{0}}\times 100$$

where *W*_0_ is the initial weight of the sample, and *W_f_* is the weight of the dried material after 24 h of solubilization.

### 2.7. ATR-FTIR Spectroscopy

The Fourier transform infrared (FTIR) spectra of the film samples were recorded with an FTIR spectrometer (Bruker, Karlsruhe, Germany) over a range of 400 to 4000 cm^−1^, with a spectral solution of 1 cm^−1^. The instrument was operated in the attenuated total reflectance (ATR) mode.

### 2.8. Color Measurement

The color parameters of the film were measured using a spectrophotometer (Ultrascan Pro, Hunterlab). The color values of the films were analyzed according to the CIE L*a*b* system (L* (lightness), a* (green to red), and b* (blue to yellow)). The C* value specifying chroma (vividness/dullness) and *h°* denoting hue angle was calculated from the a* and b* values. The yellowness index (defined by the ASTM E313 standard [18]) and haze (defined by the ASTM D1003-13 standard [19]) were also reported.

### 2.9. Measurement of Water Contact Angle

The water contact angle of the film was measured using a tensiometer (Theta Lite TL100 (Biolin Scientific, Stockholm, Sweden). Subsequently, 10 μL of distilled water was dispensed from a syringe onto the surface of the film. The static water contact angle was then determined by averaging the measurements from both the left and right sides of the droplet after 5 s, by which point the droplet had stabilized on the surface.

### 2.10. Antioxidant Properties of the Films

The antioxidant properties of the films were analyzed through the DPPH assay. To conduct this analysis, the film was dissolved in 5 mL of ethanol, and then 3 mL of a DPPH solution (0.008% *w/v* in ethanol) was introduced into the film solution and mixed well. The resulting mixture was kept in the dark for 30 min. Subsequently, the absorbance value at a wavelength of 517 nm was measured using a UV-visible spectrophotometer (SPECORD PLUS, Analytik Jena, Jena, Germany). The DPPH radical scavenging activity (AA) of the films was then calculated as follows:

(2) $$AA(\%)=\frac{A_{DPPH}-A_{sample}}{A_{DPPH}}\times 100$$

where *A_DPPH_* is the absorbance of the control, and *A_sample_* is the absorbance of the sample.

### 2.11. UV-Blocking Efficiency Test

UV protection properties can be quantified using UV Protection Factor (UPF) values, which are commonly used for fabrics [20]. The UPF represents the ratio of the average effective UV radiation irradiance transmitted through the air to the average effective UV radiation irradiance transmitted through the sample. This value can be determined using the following equation:

(3) $$UPF=\frac{\sum_{200nm}^{400nm}E_{\lambda }\times S_{\lambda }\times \Delta \lambda }{\sum_{280nm}^{400nm}E_{\lambda }\times S_{\lambda }\times T_{\lambda }\times \Delta \lambda }$$

where *E_λ_* is the relative erythemal spectral effectiveness, *S_λ_* is the solar spectral irradiance (W m^−2^ nm^−1^), Δ*λ* is the measured wavelength interval (nm), and *T_λ_* is the average spectral transmittance of the fabric.

In this work, the UPF rating was used to assess the UV-blocking efficiency of edible films. The measurements were conducted using a UV-visible spectrophotometer (Camspec M550SPF double beam scanning spectrophotometer, Spectronic Camspec Ltd., Leeds, UK). The average of three values was reported following the AATCC TM183:2020 standard [21].

### 2.12. Water Vapor Transmission Rate

The water vapor transmission rate (WVTR) was measured in accordance with ASTM F1249 [22] using a MOCON Permatran 3/34 module (Ametec Inc., Minneapolis, MN, USA). A sample film (2 cm × 2 cm) was used. The test was conducted on an effective area of 5 cm^2^ under specific conditions, i.e., a temperature of 37.8 °C and a relative humidity of 75%.

### 2.13. Application of Edible Film Packaging on Apple

An entire apple was immersed in the film solution, and both coated and uncoated apples were placed in an incubator (Binder, KBF115, BINDER GmbH, Tuttlingen, Germany) set at 25 °C for 21 days. The percentage weight loss was calculated according to the following equation:

(4) $$\mathrm{Weight}\;\mathrm{loss}\;(\%)=\frac{W_{i}-W_{f}}{W_{i}}\times 100$$

where *W_i_* is the initial weight of the sample, and *W_f_* is the weight of the incubated sample.

### 2.14. Evaluation of pH Sensing Capability of the Films

To investigate the effect of various solution pH levels on the color of SWC2, a rectangular film (1 cm × 1 cm) was wetted with distilled water at pH values ranging from 3 to 13 (adjusted using NaOH and HCl).

To investigate the sensitivity of the films to ammonia vapor, a SWC2 film (2 cm × 2 cm) was placed in direct contact with ammonia vapor, which was generated from a 30% ammonium hydroxide. The test was concluded when a color change was observed. Subsequently, the sample was left under ambient conditions before the test was repeated to assess the reusability of the indicator film.

## 3. Results and Discussion

### 3.1. Physical Appearance

**Figure 1:**
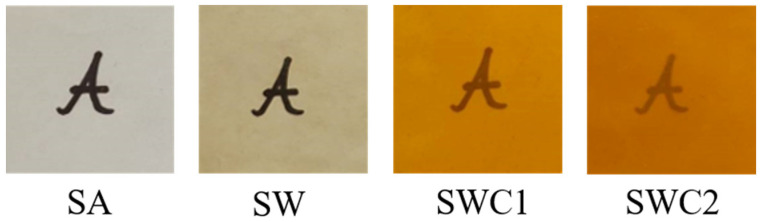
Photographic images of the films placed on top of a black “A” character.

The thickness values of SA, SW, SWC1, and SWC2 were 0.12 ± 0.01 μm, 0.15 ± 0.01 μm, 0.17 ± 0.01 μm, and 0.17 ± 0.02 μm, respectively. The introduction of WPI and CUR resulted in an increase in film thickness. From the images in Figure 1, the SA film exhibited a slight brown hue, with a corresponding yellowness index of 37.67 and a haze of 18.03% (refer to Table 1). The films containing curcumin (SWC1 and SWC2) exhibited a significant increase in the b* value (a higher positive b* value indicates a more yellow shade), as well as a higher yellowness index and haze. The increased haze in SW, SWC1, and SWC2 compared to SA indicated that both WPI and CUR contributed to reduced film transparency due to light scattering and absorption. Given the yellow pigmentation of CUR, this outcome was expected as it introduced yellowness and opacity to the film. It is worth noting that the inclusion of protein was previously reported to decrease film transparency, as reported by Lalnunthari et al. [23]. They suggested that the combination of whey protein with pectin and alginate resulted in the formation of polysaccharide–protein aggregates, leading to a film with higher opacity, lower gas barrier, lower water affinity, and denser structures due to the polysaccharide–protein interaction [24]. Importantly, the films created in our study displayed macroscopic homogeneity.

**Figure 2:**
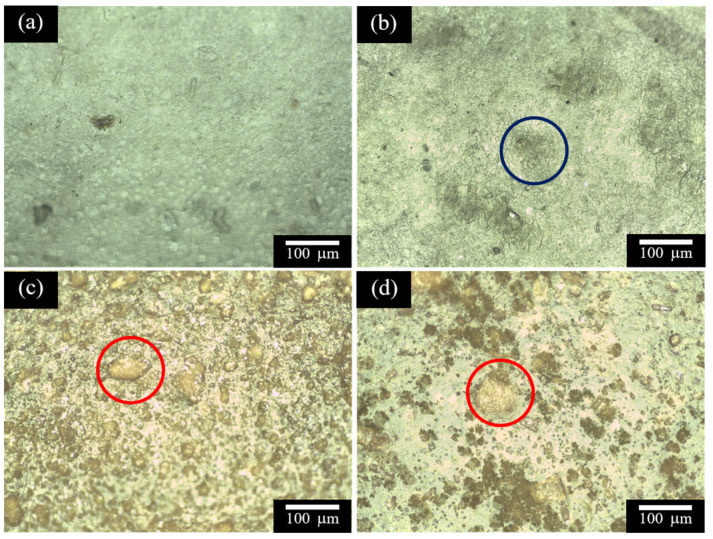
Optical micrographs of (**a**) the SA film, (**b**) the SW film, (**c**) the SWC1 film, and (**d**) the SWC2 film. The blue circle in part (**b**) represents WPI, and the red circles in (**c**,**d**) represent CUR.

The surface topographies of the films are revealed by the optical micrographs in Figure 2. Here, the SA film exhibits a smooth and regular surface with only small imperfections (Figure 2a). By comparison, the surface of the SW film is less smooth due to the presence of WPI as a separate phase (blue circle, Figure 2b). This indicates that SA is not miscible with WPI. Meanwhile, the SWC1 and SWC2 films (Figure 2c,d) display many surface irregularities with a high degree of surface roughness. This is due to the presence of CUR aggregates dispersed throughout the films (e.g., the red circles).

It is worth noting that the SA film exhibited high hygroscopicity, resulting in stickiness when stored under ambient relative humidity or when handled with bare hands. However, with the incorporation of either WPI or CUR, the film surface remained dry and non-sticky, even when stored under ambient conditions.

### 3.2. Water Solubility and Swelling Test

All the films underwent crosslinking with CA to enhance their water stability. However, it is worth noting that partial dissolution of the films was still observed, which is typical for CA-crosslinked polysaccharides [25,26]. Excessive CA content may not be advantageous, as it has been reported that an excess of CA resulted in a higher number of hydroxyl groups, which could lead to high water solubility [25].

As indicated in Table 2, SA exhibited high water solubility in comparison to the other films. The incorporation of both WPI and CUR contributed to improved water resistance in the films, with CUR having a more pronounced effect. Films with a higher CUR content exhibited lower water solubility, with SWC2 demonstrating the lowest water solubility among the films. This enhanced water stability can be attributed to the hydrophobic properties of CUR (the water solubility of curcumin is limited to 0.6 μg/mL [27].

In the context of the swelling test results, the introduction of WPI and CUR to the films led to an increase in the swelling degree when placed in water. This can be attributed to the films with WPI or CUR being capable of retaining more water. In contrast, films without CUR and WPI exhibited high solubility and, as a result, disintegration in water instead of swelling.

### 3.3. Fourier Transform Infrared Spectroscopy (FTIR)

**Figure 3:**
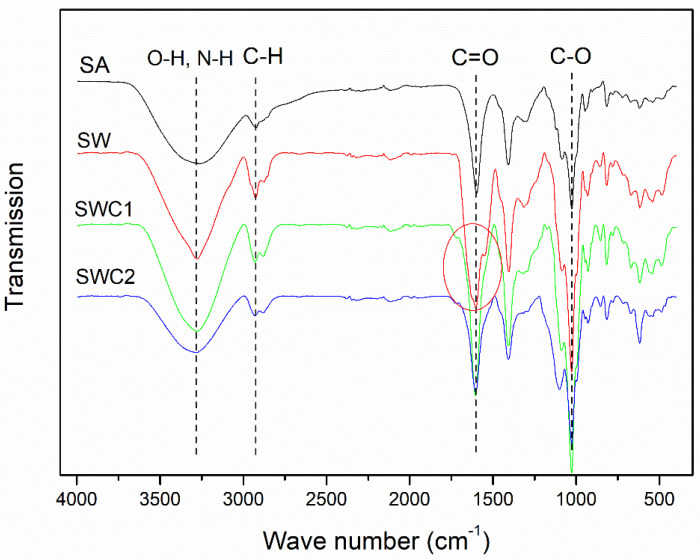
ATR–FTIR spectra of the SA, SW, SWC1, and SWC2 films.

Figure 3 shows the FTIR spectra of the edible films. All the FTIR spectra exhibit a broad and strong peak in the range of 3000–3600 cm^−1^, which is characteristic of O–H stretching associated with hydroxyl groups. However, in the SW film, there is a medium N–H stretching peak at around 3300 cm^−1^, overlapping with the O–H stretching peak. Notably, this N–H stretching peak is less prominent in samples containing CUR despite their structural inclusion of hydroxyl groups. It is possible that the presence of CUR at the surface of the film may have masked the WPI, leading to the detection of only CUR by ATR-FTIR. The absorption band associated with C–H stretching falls within the 2927–2930 cm^−1^ range, observed in all samples. The peak associated with the amide group in WPI is evident at 1604 cm^−1^ (representing the stretching of C=O, corresponding to amide band I) and at 1407 cm^−1^ (attributable to vibration within the plane of C–N). It is worth noting that the C=O peak around 1600 cm^−1^ can also be associated with carbonyl groups present in SA (C=O of the ester group) and CUR (C=O of the ketone group), nearly coinciding with the C=O of the amide groups in WPI. Typically, the C=O stretching peak of the amide group is slightly shifted to lower wave numbers than the C=O stretching of the ester group. Consequently, a subtle split of the C=O peak is observed in the SW sample due to the coexistence of SA and WPI. The absorption band at 1027 cm^−1^ is related to C–O stretching vibrations and is consistent across all samples. Overall, the addition of WPI or CUR to the alginate films only results in minor variations in the appearance of the FTIR spectra.

### 3.4. Water Contact Angle

**Figure 4:**
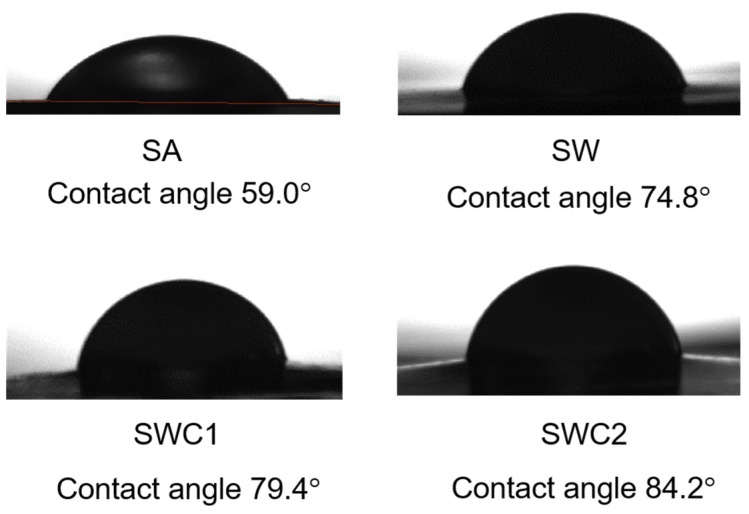
Water contact angle measurement results.

Figure 4 shows the images of the water droplets on the surface of the films. If the contact angle is less than 90 degrees, the film is considered hydrophilic. Therefore, a lower contact angle indicates a higher degree of hydrophilicity for the films. It is evident from the images that all alginate-based films exhibited hydrophilic properties. However, the inclusion of WPI and CUR further reduced the hydrophilicity of the films. This can be attributed to the lower polarity of WPI and CUR compared to that of pristine alginate.

### 3.5. Antioxidant Properties of the Films

Active food packaging with antioxidant properties can effectively prolong the shelf life of food products by inhibiting oxidative reactions initiated by free radicals [28]. CUR is well-known for its exceptional antioxidant capacity, effectively neutralizing various reactive oxygen species, including hydroxyl radicals and nitrogen dioxide radicals. CUR achieves this by scavenging reactive radicals and engaging in antioxidant activities, primarily through the donation of H-atoms from its phenolic O–H group [29]. In the scope of this study, an in vitro antioxidant DPPH assay was conducted to assess the DPPH scavenging capacity of the edible films. While SA and WPI have been recognized for their antioxidant properties, they do not exhibit the same level of antioxidant potency as CUR. As demonstrated in Table 3, it is evident that in the absence of CUR, the antioxidant efficiency was notably low, achieving less than 20% efficiency. The introduction of WPI into the alginate film led to a modest improvement in scavenging efficiency. Importantly, as the CUR content increased, the film displayed an enhanced antioxidant efficacy, reaching an antioxidant activity level of 82.39% for SWC2.

### 3.6. UV-Blocking Efficiency Test

**Figure 5:**
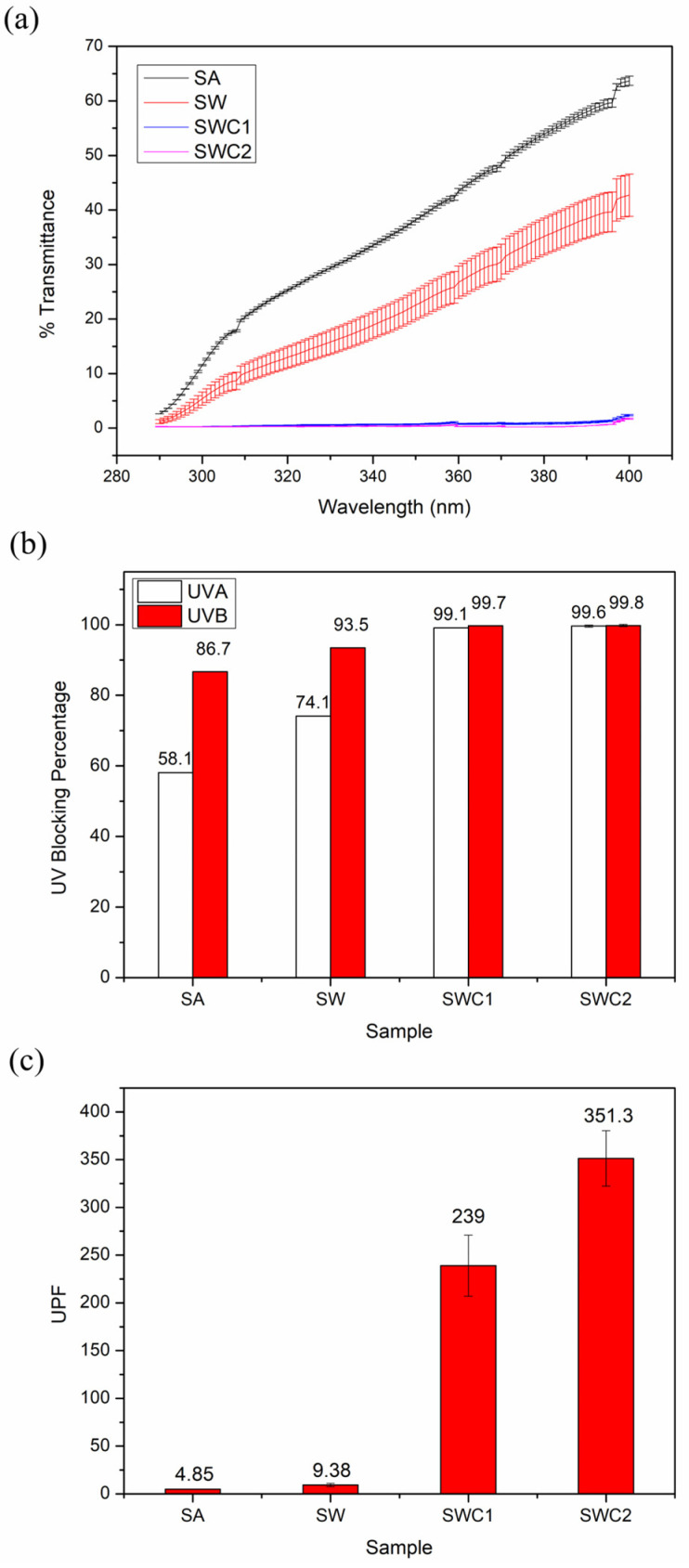
UV protection properties of edible films: (**a**) transmittance versus wavelength, (**b**) UV-blocking percentages, and (**c**) UPF values.

A UV barrier film can prevent degradation in food quality induced by photo-oxidation. Foods that are highly sensitive to photo-oxidation include dairy products, nuts, meat, and wine [30]. Figure 5 details the UV transmittance values, UV-blocking percentages, and UPF ratings of the prepared edible films. The presence of WPI and CUR in the films significantly improved UVA-(wavelength 315–400 nm) and UVB-(wavelength 280–315 nm) blocking efficiency, with an emphasis on UVB. The SA film exhibited UVA- and UVB-blocking efficiencies of 58.1% and 86.7%, respectively. In contrast, the SW film resulted in higher values, reaching 74.1% for UVA and 93.5% for UVB. Remarkably, SWC1 and SWC2 achieved nearly 100% efficiency for both UV regions. It is worth noting that a UPF rating of 50 and above is considered excellent for UV protection applications [31]. The UV-blocking efficiency of WPI and CUR can be attributed to a combination of light scattering and absorption, a phenomenon that aligns with the increased opacity of the films in the presence of WPI and CUR. Additionally, CUR effectively absorbs UV light due to its molecular structure, which contains conjugated π electrons, including adjacent C=C bonds linked to carbonyl groups and phenolic moieties.

### 3.7. Water Vapor Transmission

In food preservation, it is generally imperative to minimize the exchange of moisture between food and its surrounding environment to maintain the quality of food products [32,33]. The WVTR measures the rate at which water vapor passes through the film. Films with low WVTR are particularly promising for packaging fruits, as they help to mitigate moisture loss from the fruits. The WVTR values for the prepared films are detailed in Table 4.

The SA film exhibited poor water barrier properties due to the hydrophilic nature of the polymer. The WVTR of the SA film was the highest among the prepared films, with a WVTR of 569.44 ± 32.14 g·m^−2^·day^−1^. Nevertheless, this value was lower than that of the SA/hydrolyzed collagen film (WVTR = 1023.4 ± 121.9 g·m^−2^·day^−1^) [33] and SA crosslinked with CaCl_2_ (WVTR > 2000 g·m^−2^·day^−1^) [34]. Notably, chemical crosslinking with citric acid enhanced the water barrier properties of the film. Overall, these results indicated that the SA film did not function effectively as a water barrier. However, SA played a role as a sacrificial moisture agent, allowing moisture to evaporate from the film rather than from the food surface [2].

The influence of WPI on WVTR was not readily discernible, as the WVTR value for the SW film closely resembled that of the SA film. However, the incorporation of CUR into the film improved the water barrier properties due to the CUR pigment increasing the tortuosity and hydrophobicity of the film. This led to significantly lower WVTR values for SWC1 and SWC2 films compared to the SA film. Consequently, it was anticipated that CUR-containing films would be more effective in preventing moisture loss from food.

### 3.8. Mechanical Properties

The mechanical properties of the films are revealed by the tensile test results in Table 5. The tensile strengths of the SW, SWC1, and SWC2 are 37.25 ± 5.30, 18.64 ± 4.82, and 17.39 ± 3.25 MPa, respectively. These significant reductions in tensile strength compared to the SW can be attributed to the non-uniformity of the CUR-loaded films. This is consistent with the OM images in Figure 2, where the immiscible CUR was seen to form aggregates in the film matrix, thereby creating defect sites. Meanwhile, the SW, SWC1, and SWC2 exhibit elongation at break values of 34.74 ± 7.99, 53.8 ± 3.46, and 37.11 ± 3.82%, respectively. Notably, the film with a low CUR concentration (SWC1) exhibits a higher elongation at break than either the non-CUR film (SW) or the film with a high CUR content (SWC2). This indicates that CUR increases the film flexibility but also weakens the bonds between the alginate molecules. Moreover, the higher CUR loading increases both the size and amount of CUR aggregates, thereby forming a more defective and highly brittle film. Thus, too high a CUR content negatively affects both the strength and flexibility of the film. This observation agrees with a previous study on CUR-loaded proso millet-derived starch films, where the authors reported that increasing the concentration of CUR (0–3%) decreased the tensile strength but increased the elongation at break of the films [35].

### 3.9. Application of Edible Film Packaging on Apple

**Figure 6:**
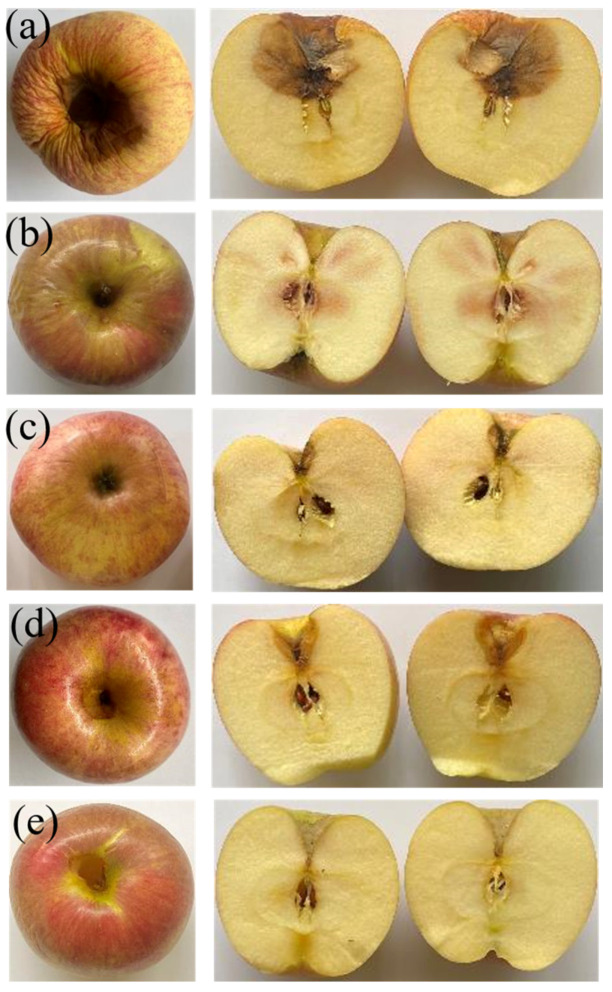
Photographic images of the incubated apples: (**a**) uncoated apple; (**b**) SA-coated apple; (**c**) SW-coated apple; (**d**) SWC1-coated apple; and (**e**) SWC2-coated apples.

**Figure 7:**
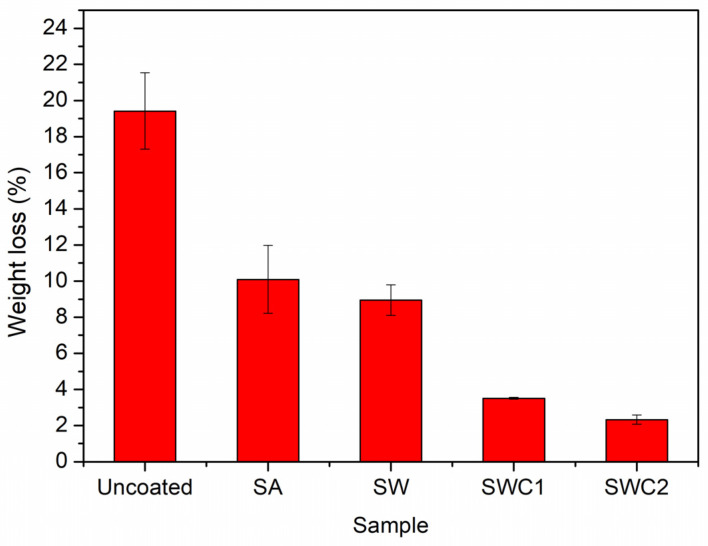
Weight loss percentages of incubated apples with and without coatings.

Apples continue to respire even after being harvested, a process in which glucose is converted into carbon dioxide and water, which results in moisture and weight loss. Reducing the respiration rate of apples is crucial, and this can be achieved by minimizing the exchange of oxygen, carbon dioxide, and moisture with the surrounding environment. Furthermore, lowering the respiration rate also plays a role in partially inhibiting enzymatic browning, which is associated with reduced ethylene production [4]. Figure 6 shows whole apples before and after a 21-day incubation period at 25 °C. Untreated apples experienced nearly a 20% loss in their original weights, while the coated apples demonstrated more effective weight preservation (see Figure 7). As evidenced by the results of water solubility and contact angle tests, films containing WPI and CUR exhibited higher hydrophobicity compared to the SA film. Consequently, apples coated with SW, SWC1, and SWC2 films were more successful in retaining their water content, resulting in less weight loss. Notably, coating with SWC2 yielded the highest residual weight, with a mere 2.33 ± 0.26% reduction. Enzymatic browning was more pronounced in SA-coated apples, followed by SW-coated apples. In summary, apples coated with films containing a high CUR content were able to maintain their freshness longer.

### 3.10. pH Sensitivity of the Films

**Figure 8:**
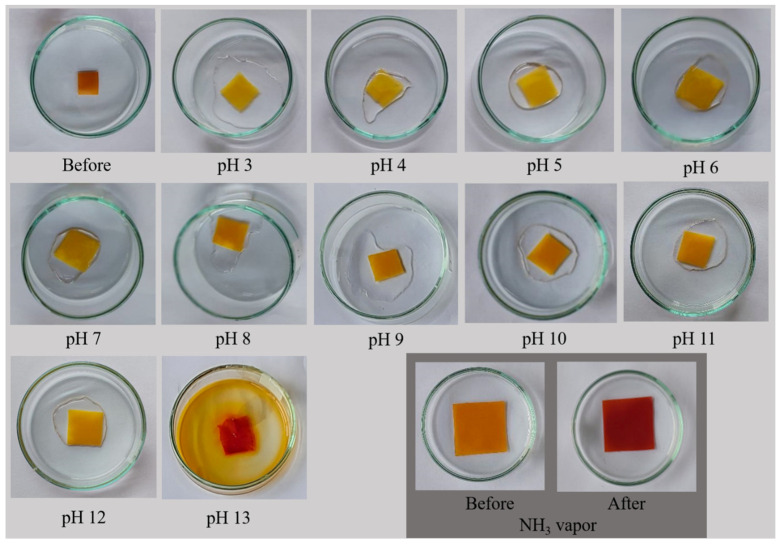
Photographic images of the SWC2 films tested with aqueous solutions at different pH values of 3 to 13, and with ammonia vapor.

CUR has found applications as a bio-based indicator in smart food packaging systems. It is well-documented that CUR undergoes a color shift from yellow within a pH range of 1 to 7, transitioning to a reddish-brown hue at a pH of 8 [36]. In this study, the SWC2 film was subjected to aqueous solutions with varying pH values ranging from 3 to 13. A subtle change in color, shifting from yellow to orange, was observed when the pH increased from 7 to 8 (see Figure 8). The color change was difficult to detect visually, likely due to CUR being in a solid state. However, when the film disintegrated in a highly alkaline solution at pH 13, CUR leached out concurrently with a noticeable color shift from yellow to red. This drastic change in color occurred because CUR rapidly degraded in the alkaline environment [37]. Remarkably, the SWC2 film exhibited the capability to sense ammonia vapor, displaying a color shift from orange to red. It required approximately 20 s of exposure to ammonia vapor for the complete color change to occur. Once removed from the ammonia vapor, the film gradually reverted to its original color. Importantly, the films were found to be reusable, rendering SWC2 a valuable candidate for smart food packaging designed for freshness monitoring. Ammonia sensing holds significance for packaging meat products such as fish, shrimp, and beef, as protein and amine degradation results in the production of total volatile basic nitrogen (TVB-N). CUR-incorporated packaging materials have demonstrated effectiveness in monitoring shrimp spoilage [38,39,40].

## 4. Conclusions

This study investigated the influence of WPI and CUR on alginate-based edible coatings and films. Both WPI and CUR reduced the film transparency. CUR imparted a yellow hue to the films and reduced their hydrophilicity, leading to a higher water contact angle, lower water solubility, and a decreased water vapor transmission rate, all of which are favorable attributes for edible coating and film applications. Both WPI and CUR enhanced the antioxidant properties of the films, but CUR had a stronger influence. Furthermore, CUR significantly enhanced the UV-blocking properties of the films and extended the shelf life of apples. Apples coated with CUR-containing coatings exhibited significantly less weight loss and retained their freshness more effectively compared to uncoated apples. Notably, the CUR-containing films exhibited a reversible color change from orange to red when exposed to ammonia vapor. In summary, the edible coatings and films developed in this study hold significant promise as smart food packaging materials.

## Figures and Tables

**Table 1:** Color parameters and haze of the prepared films.

Sample	L*	a*	b*	C*	*h^o^*	Yellowness Index	Haze (%)
SA	91.55 ± 1.41	−0.99 ± 0.25	16.27 ± 0.86	16.30 ± 0.86	93.49 ± 0.98	37.67 ± 0.20	18.03 ± 0.56
SW	92.85 ± 1.11	−0.93 ± 0.13	15.51 ± 0.54	15.53 ± 0.55	93.43 ± 0.44	25.98 ± 0.14	50.01 ± 0.40
SWC1	68.84 ± 1.13	14.34 ± 0.71	96.55 ± 0.59	97.61 ± 0.69	81.55 ± 0.36	127.74 ± 0.38	61.34 ± 0.42
SWC2	51.52 ± 1.20	30.28 ± 0.67	86.40 ± 0.88	91.55 ± 0.99	70.69 ± 0.31	129.84 ± 0.50	63.67 ± 0.33

**Table 2:** Water solubility and swelling degree of the prepared films.

Sample	Water Solubility (%)	Swelling Degree
SA	75.55 ± 3.85	745.33 ± 6.11
SW	60.44 ± 1.84	887.47 ± 2.60
SWC1	34.22 ± 1.17	1284.67 ± 2.60
SWC2	16.67 ± 3.79	1526 ± 2.23

**Table 3:** DPPH radical scavenging activity (AA) of the films.

Sample	AA (%)
SA	12.54 ± 0.88
SW	19.65 ± 0.27
SWC1	60.07 ± 0.07
SWC2	82.39 ± 0.39

**Table 4:** WVTR values of the films (measured according to ASTM F1249).

Sample	WVTR (g·m^−2^·day^−1^)
SA	569.44 ± 32.14
SW	533.75 ± 27.89
SWC1	452.75 ± 9.47
SWC2	454.49 ± 6.76

**Table 5:** Tensile test results.

Sample	Tensile Strength (MPa)	Elongation at Break (%)
SW	37.25 ± 5.30	34.74 ± 7.99
SWC1	18.64 ± 4.82	53.8 ± 3.46
SWC2	17.39 ± 3.25	37.11 ± 3.82%

## Data Availability

Data are contained within the article.
